# Coral-like Co_3_O_4_ Decorated N-doped Carbon Particles as active Materials for Oxygen Reduction Reaction and Supercapacitor

**DOI:** 10.1038/s41598-018-19347-5

**Published:** 2018-01-29

**Authors:** Zhichao Lin, Xiuwen Qiao

**Affiliations:** Key Laboratory for Green Processing of Chemical Engineering of Xinjiang Bingtuan, State Key Laboratory Cultivation Base Jointly Constructed by Province and The Ministry, Key Laboratory of Materials-Oriented Chemical Engineering of Xinjiang Uygur Autonomous Region, College of Chemistry and Chemical Engineering, Shihezi University, Shihezi, 832000 China

## Abstract

Coral reef has a unique dendritic structure with large specific surface area, rich pore structure, so that it can be attached to a large number of zooxanthellae for gas exchange. Coral reef ecosystems are also known as underwater rainforests. Inspired by this biological structure, we designed and fabricated coral-like Co_3_O_4_ decorated N-doped carbon particles (Co_3_O_4_/N-CP). The obtained Co_3_O_4_/N-CP-900 catalyst shows efficient ORR electrocatalytic performances in an alkaline medium with a positive onset and half-wave potentials of 0.97 and 0.90 V (vs. RHE), as well as a high diffusion-limited current density (5.50 mA cm^−2^) comparable to that of a Pt/C catalyst (5.15 mA cm^−2^). It also displays better stability and methanol tolerance than commercial Pt/C. In addition, the Co_3_O_4_/N-CP-900 electrode has a high specific capacitance of 316.2 F g^−1^ in 6 M KOH, as well as good rate capabilities and excellent cycle performance. These results are due to large surface area, narrow pore size distribution, high density electrochemical energy conversion and storage activity centers. This method presented here offers an effective path for the development of high performance multi-functional carbon-based materials for ORR and supercapacitor applications.

## Introduction

The development of new energy sources, such as fuel cells and supercapacitors, is the key to reduce the consumption of traditional energy. Therefore, numerous efforts have been devoted to the development of electrode catalysts for energy conversion and storage^1,2^, in order to promote the commercialization of fuel cells and supercapacitors. Generally, platinum and its alloy catalysts are considered to be the most effective Oxygen Reduction Reaction (ORR) catalyst^3,4^. However, they suffer from the expensive, limited resources, poor stability, which hinder its application in practice^5^. Due to the low cost and good stability of the heteroatom-doped carbon materials, they can be used as an ideal material for fuel cells^6,7^ and supercapacitors^8^. Recent studies have shown that the ORR activity of heteroatom-doped carbon materials is due to the redistribution of charge induced by doping around the heteroatom dopant, which reduces the ORR potential and changes the chemical adsorption of O_2_, effectively weakening O-O bonding^9–12^. At the same time, heteroatom-doped carbon materials can be used as an electrode material for the supercapacitors, the introduction of heteroatoms in the carbon material can increase pseudocapacitance and improve the surface wettability, which is conducive to improving the performance of supercapacitors^13,14^. Despite their considerable development, performance optimization is still needed to ensure that heteroatom-doped carbon materials can simultaneously satisfy requirements of ORR and supercapacitor.

In order to obtain efficient multi-functional materials for ORR and supercapacitors, the introduction of transition metal oxides (e.g., Co, Fe) into carbon materials doped with heteroatoms (e.g., N, S) may lead to electron modulation to provide an ideal electronic structure for relatively good electrocatalytic activity, which is considered to be an effective method^15,16^. At the same time, we believe that the high specific surface area and narrow pore size distribution are also the key to determine the performance^17–19^. In nature, polyps adsorb iron, manganese and other elements during their formation, eventually forming a dendritic coral that is rich in pore structure. A kind of zooxanthella attached to the surface of the coral, converting the metabolic waste into O_2_ and carbohydrate by photosynthesis, and then returned to the polyps (Fig. S1). Due to the high specific surface area, rich pore structure and gas exchange by the attachment of a large amount of zooxanthellae, coral reef ecosystems are also known as underwater rainforest^20–22^. Inspired by this biological structure, we have tried to create a coral-like carbon structure that has a large specific surface area, abundant porous structure and incorporates the Co element on the surface. The structure acts like zooxanthellae to construct electrochemical energy conversion and store active center, which is conducive to the application of ORR and supercapacitors. This structure was similar to the symbiotic system of polyps and zooxanthellae, expected to improve the electrochemical performance of carbon-based materials.

Herein, we have developed a simple, effective strategy for the preparation of the coral-like Co_3_O_4_ decorated N-doped carbon particles (Co_3_O_4_/N-CP-X) (X represents as temperature) for the first time. Co_3_O_4_/N-CP-X has been prepared through self-assembly of polyaniline (PANI) as the precursor, hydrothermal synthesis of Co_3_O_4_, and then pyrolysis, which displayed efficient catalytic activity for oxygen reduction and excellent capacitive properties in an alkaline solution (Fig. S2). In particular, the Co_3_O_4_/N-CP-900 acted as ORR electrocatalyst showing the positive onset and half-wave potentials (0.97 V and 0.90 V), basically compared to Pt/C catalyst (0.99 V and 0.89 V), and displayed high stability, good methanol tolerance in alkaline solution. Moreover, Co_3_O_4_/N-CP-900 can be used as electrode material for supercapacitor, with a high specific capacitance of 316.2 F g^−1^ at a current density of 1 A g^−1^, as well as long-term stability, good rate capabilities and excellent cycle performance. The improved electrochemical properties can be attributed to the high surface area, narrow pore size distribution and multi-element doping of the coral-like structure, which provides higher density of active sites, better obtaining electrolyte, greater ion storage space, faster electrolyte diffusion and movement for Co_3_O_4_/N-CP-X. This work provides an effective way to produce heteroatom-doped carbon materials for electrochemical energy conversion and storage.

## Results and Discussion

### Synthesis of Co_3_O_4_/N-CP-X

**Figure 1:**
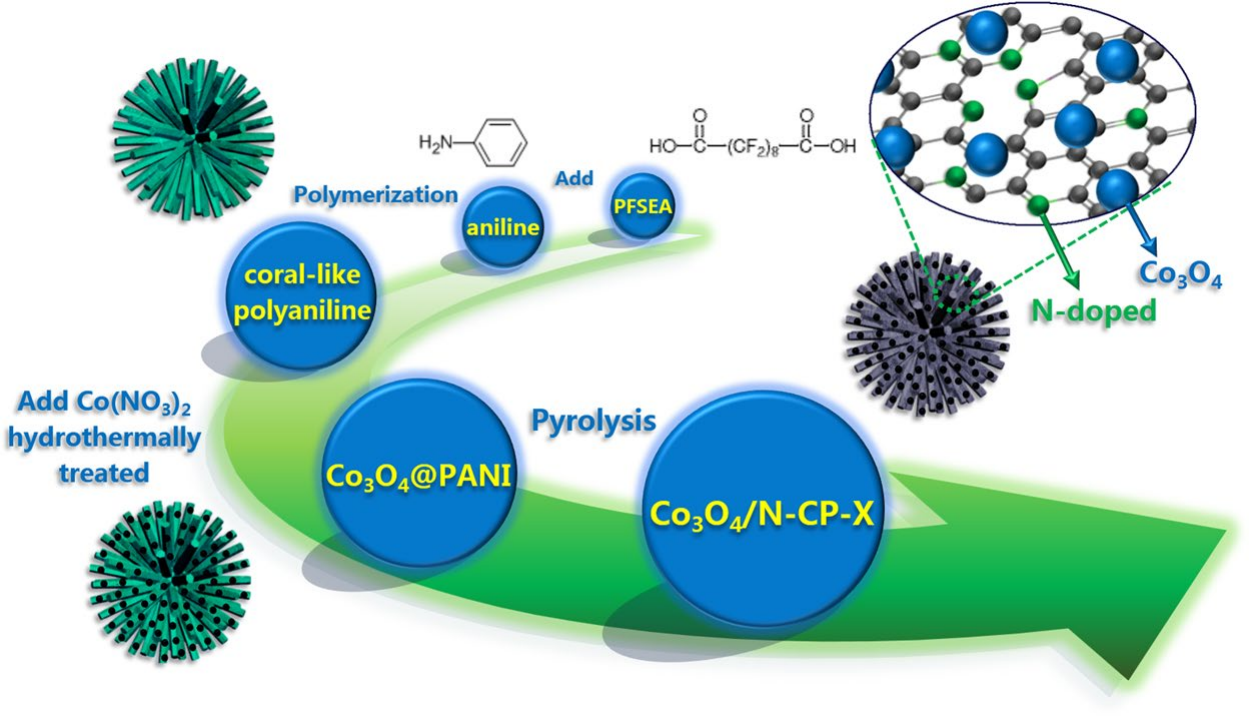
Scheme illustration of self-assembly with PANI and the derived Co_3_O_4_/N-CP-X.

The typical synthetic steps of coral-like Co_3_O_4_/N-CP-X showed in Fig. 1. The coral-like polyaniline doped with perfluorosebacic acid (PANI/PFSEA) precursor was fabricated by chemical polymerization in the presence of aniline, PFSEA and Co(NO_3_)_2_·6H_2_O acted as monomer, dopant and oxidant, respectively. Then coral-like Co_3_O_4_/N-CP-X particles (X represents pyrolysis temperature) were prepared by hydrothermal treatment of PANI/PFSEA and Co(NO_3_)_2_·6H_2_O at 180 °C, thus leading to crystallization of Co_3_O_4_ and reduction of PANI to form the Co_3_O_4_/PANI hybrid^23^, then followed by pyrolysis in tubular furnace at 800 °C, 900 °C, 1000 °C under the nitrogen atmosphere for 3.0 h with a heating rate of 5 °C/min, respectively. For comparison, the coral-like N-doped carbon materials (referred to as N-CP-X) were also prepared by the same procedure described above for Co_3_O_4_/N-CP-X, except for addition cobalt nitrate during the hydrothermal process.

### Structural and microstructural analyses of Co_3_O_4_/N-CP-X

**Figure 2:**
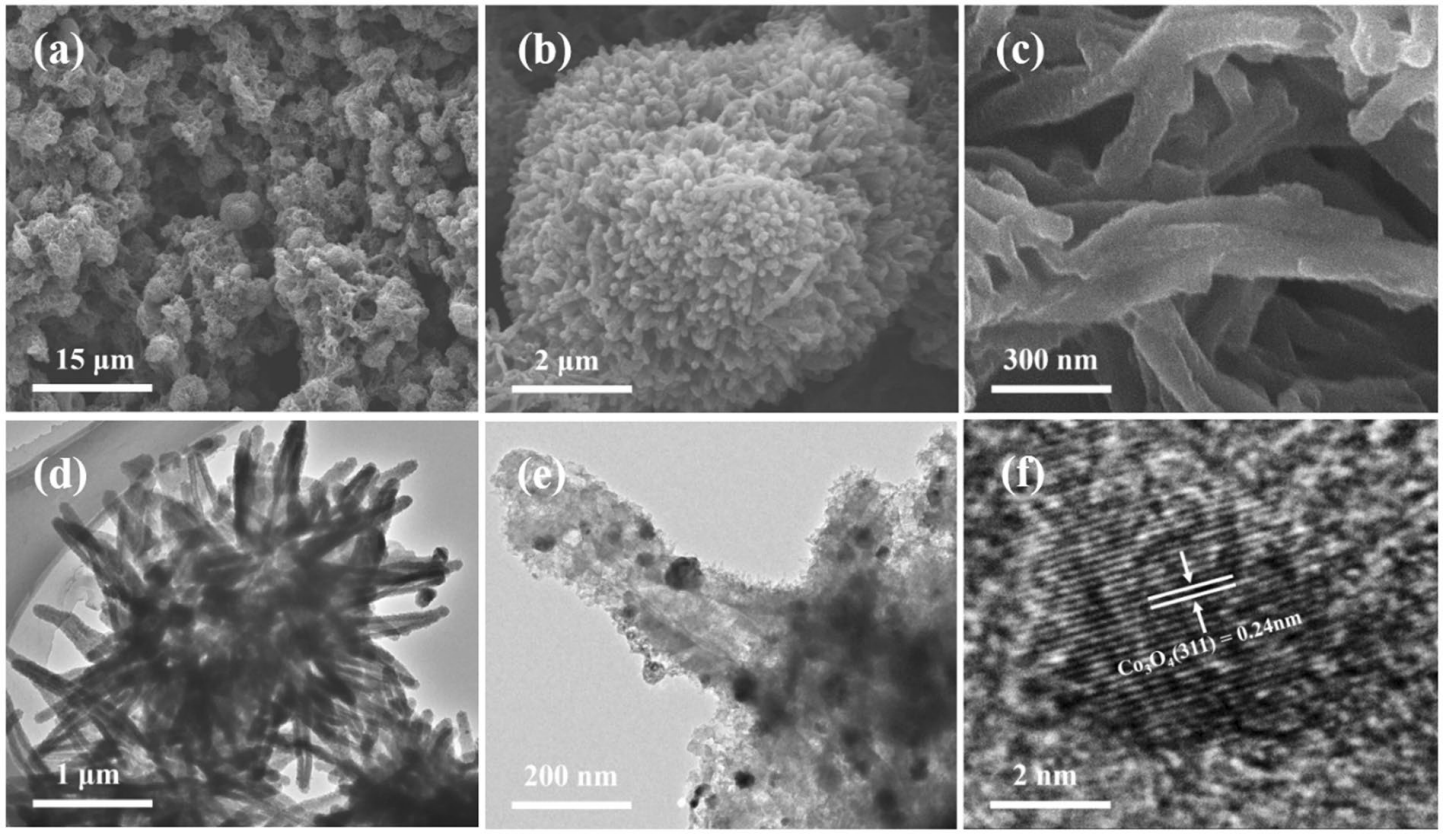
(**a**–**c**) SEM images, (**d**–**e**) TEM images, and f) HRTEM images of coral-like Co_3_O_4_/N-CP-900.

Fig. 2a–c showed the SEM images of Co_3_O_4_/N-CP-900, Fig. 2d–f showed the TEM images of Co_3_O_4_/N-CP-900, Fig. S3a–d showed the SEM images of coral-like polyaniline and Co_3_O_4_/PANI, Fig. S4a–d showed the SEM images of Co_3_O_4_/N-CP-800 and Co_3_O_4_/N-CP-1000. As shown in Fig. 2, Figs S3 and S4, all of samples displayed a well-developed, defined coral-like 3D morphology and the coral-like structure of Co_3_O_4_/N-CP-X sample maintained after hydrothermal reaction and pyrolysis. Co_3_O_4_/N-CP-900 displayed a uniform and coral-like structure with a diameter of 3 μm that was composed of nanofibers with diameter ca. 180 nm (Fig. 2a–c). TEM image also confirms the above results (Fig. 2d), showed a coral-like radial structure, which is beneficial to increase the specific surface area and provide more active sites. The enlarged TEM image showed that the carbon nanotubes structures decorated some Co_3_O_4_ nanoparticles with a diameter range about from 10–50 nm (Fig. 2e). The morphology of Co_3_O_4_/N-CP-900 was further studied using high resolution transmission electron microscopy (HRTEM). The HRTEM image showed that the nanoparticles decorated on carbon nanotubes (Fig. 2f), simultaneously exhibiting a spacing of crystalline lattices of 0.24 nm and corresponding to the Co_3_O_4_ phase [311] planes^24^, which indicated high crystallinity of cobalt oxide nanocrystal.

**Figure 3:**
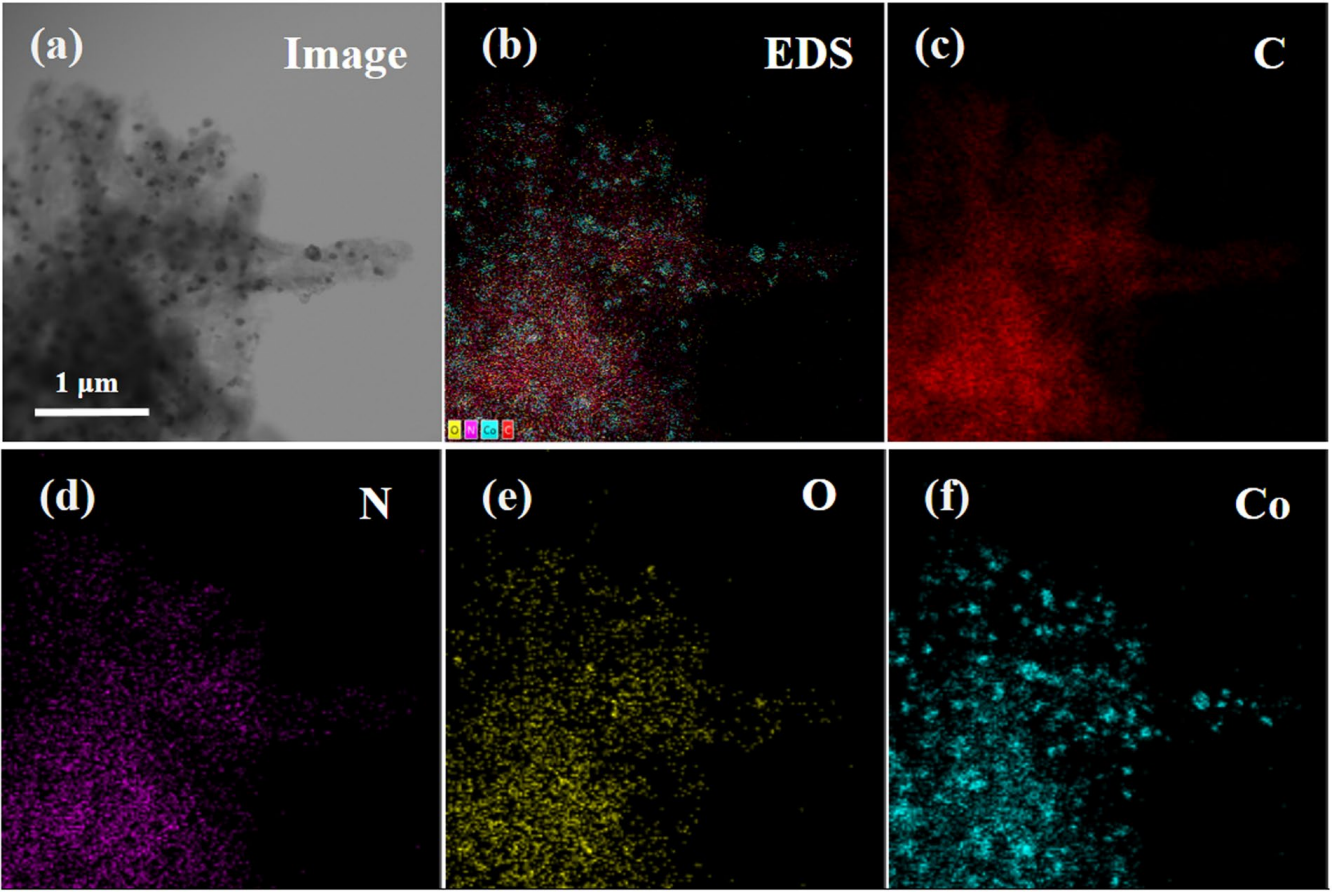
STEM and the corresponding elemental mapping images of coral-like Co_3_O_4_/N-CP-900 indicating the distribution of C, N, O and Co elements.

The scanning transmission electron microscopy (STEM) image of the coral-like Co_3_O_4_/N-CP-900 and the corresponding energy dispersive X-ray spectroscopy (EDS) elemental mapping were given in Fig. 3. STEM bright-field image (Fig. 3a) suggested that the black nanoparticles were decorated on carbon nanotubes. The EDS mapping of C, N, O and Co were shown in Fig. 3c–f, corresponding to the STEM bright-field image, further indicated that many cobalt oxide nanoparticles were decorated on carbon nanotubes of coral-like Co_3_O_4_/N-CP-900, thus providing a high density of active sites. The EDS elemental mappings of N confirmed a uniform distribution of N atoms in coral-like Co_3_O_4_/N-CP-900 particles, which is important to promote the electrocatalytic efficiency for ORR and supercapacitor. The above results verified that the Co_3_O_4_ nanoparticles anchored on N-doped carbon nanomaterials might enhance electrochemical stability of coral-like Co_3_O_4_/N-CP-900 for ORR and supercapacitor. Furthermore, the anchored metal oxide nanoparticles could generate a unique host-guest electronic interaction and change the local work function of the carbon, making the outer surface of the carbon layer more active to ORR^25^.

**Figure 4:**
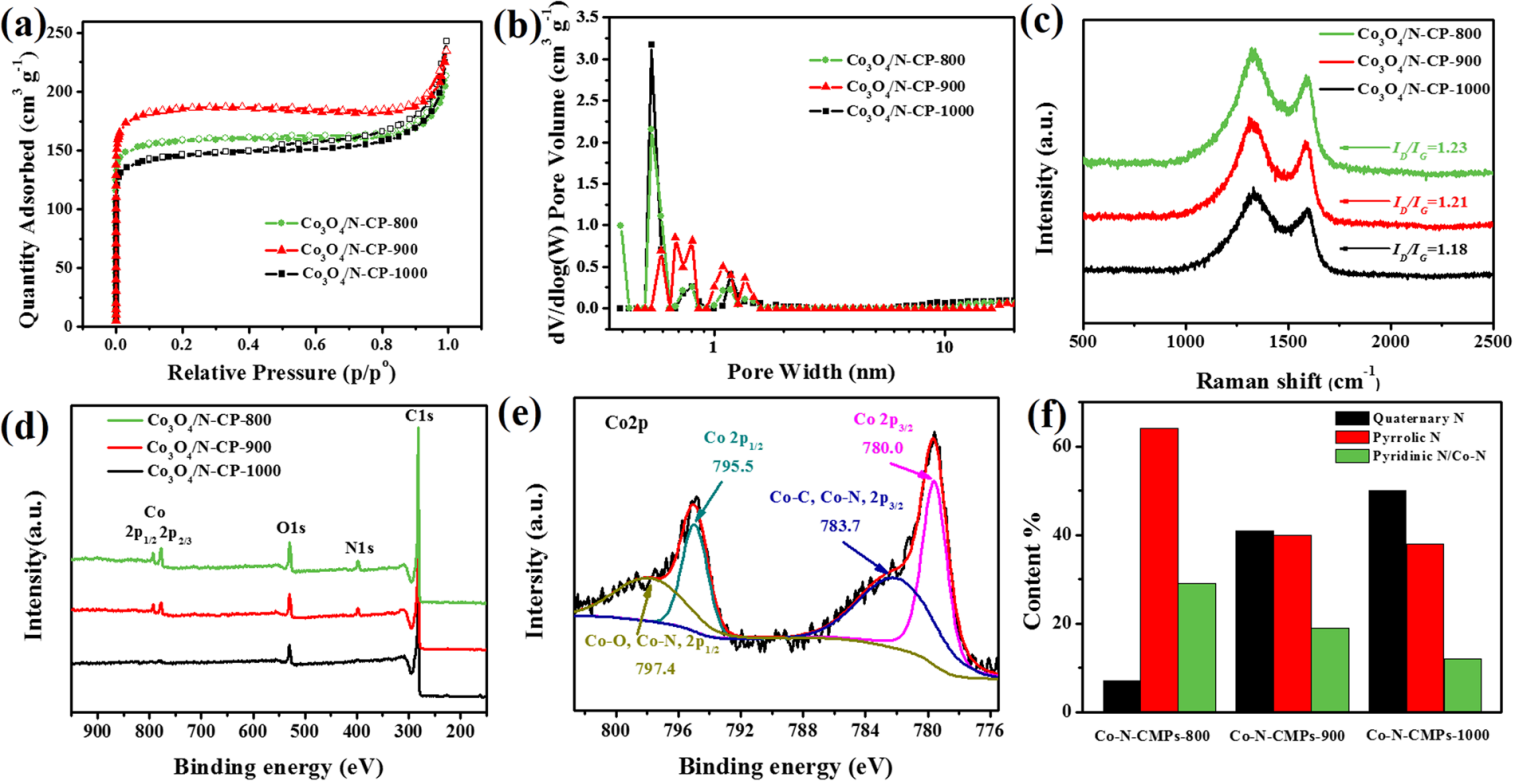
(**a**) N_2_ adsorption-desorption isotherm of the Co_3_O_4_/N-CP-X, (**b**) Corresponding pore size distribution curves of the Co_3_O_4_/N-CP-X, (**c**) Raman (λ_ex_ at 633 nm) spectra of the Co_3_O_4_/N-CP-X, (**d**) XPS survey spectra of the Co_3_O_4_/N-CP-X, (**e**) High-resolution Co2p XPS spectrum of the Co_3_O_4_/N-CP-900, (**f**) The N species distribution of the Co_3_O_4_/N-CP-900.

The coral-like Co_3_O_4_/N-CP-X materials were investigated by Brunauer-Emmett-Teller (BET) surface area, pore size distribution, Raman spectroscopy, X-ray photoelectron spectroscopy (XPS), X-ray diffraction (XRD). Brunauer-Emmett-Teller (BET) surface area and pore size distribution were investigated by a N_2_ adsorption-desorption analysis, as shown in Fig. 4a,b. All the coral-like Co_3_O_4_/N-CP-X catalysts show the type I isotherm in Fig. 4a, manifesting they are the properties of microporous materials^17^. The Co_3_O_4_/N-CP-900 exhibited the higher BET surface area of 738.3 m^2^ g^−1^ than 621.4 m^2^ g^−1^ for Co_3_O_4_/N-CP-800, and 449.5 m^2^ g^−1^ for Co_3_O_4_/N-CP-1000. It can be concluded that increasing treatment temperature from 800 to 900 °C significantly increase the specific surface area, but further increasing temperature leads to a slight decrease in the specific surface area, which may be attributed to the partial destruction of ordered micropores^18,19^. It could be considered that the high surface area materials would introduce more active sites, thereby enhancing the catalytic ORR activity and supercapacitor performance. The porous structures were observed in the pore size distribution curves of Co_3_O_4_/N-CP-X (Fig. 4b). Barrett-Joyner-Halenda (BJH) desorption average pore diameter for Co_3_O_4_/N-CP-800, Co_3_O_4_/N-CP-900 and Co_3_O_4_/N-CP-1000 were found to be 0.5, 0.6 and 0.4 nm, respectively. The peak below 1 nm observed in the pore size distribution curves for all the materials further pointed towards the majority of micropores, which facilitates oxygen adsorption and desorption, as well as exposure to active sites and rapid ion and electrolyte transport.

As shown in Fig. 4c, the Raman spectra of coral-like Co_3_O_4_/N-CP-X displayed two bands; D band and G band at 1,335 cm^–1^ and 1,580 cm^–1^, respectively, corresponding with the disordered graphitic carbon and the vibration of the sp^2^-bonded carbon atoms in the two-dimensional hexagonal lattice, which indicated the formation of graphite carbon during pyrolysis^19,26^. The integrated intensity ratio of the D and G band (I_D_/I_G_) is widely used to assess the graphite material defect density^19,26^. The I_D_/I_G_ values of Co_3_O_4_/N-CP-800 was 1.23, Co_3_O_4_/N-CP-900 was 1.21, and Co_3_O_4_/N-CP-1000 was 1.18, respectively, indicating that the graphitization degree of Co_3_O_4_/N-CP-X was improved with the increasing pyrolysis temperature. As presented in XRD patterns of Fig. S5f, the peaks are due to those of the monoclinic phase of Co_3_O_4_, which is correspond to the result of TEM.

The survey XPS spectra of coral-like Co_3_O_4_/N-CP-X showed the presence of C, O, N and Co elements (Fig. 4d), indicating that the carbon framework was successfully doped with nitrogen. We found that the nitrogen content was reduced by increasing the pyrolysis temperature due to the loss of unstable nitrogen. Nitrogen contents in Co_3_O_4_/N-CP-800, Co_3_O_4_/N-CP-900 and Co_3_O_4_/N-CP-1000 were 3.37 at %, 2.10 at % and 1.39 at %, respectively. Fig. S5a–c displayed N1s XPS spectra of the as-prepared Co_3_O_4_/N-CP-800, Co_3_O_4_/N-CP-900 and Co_3_O_4_/N-CP-1000, respectively. Deconvolution of the N1s XPS spectrum was performed with three peaks corresponding to pyridinic N (398.5 eV), pyrrolic N (400.3 eV) and graphitic-type quaternary N (401.5 eV)^27^. Since the difference between the binding energy of Co-N and pyridinic N is very small, the peak centered at 398.5 eV also includes the contribution of N bond to cobalt (Co-N)^28^. The relative N specie distributions in Co_3_O_4_/N-CP-X were compared in Fig. 4f. The ratios of graphitic-type quaternary N, pyrrole N and pyridinic N were 7%, 64% and 29% in Co_3_O_4_/N-CP-800, respectively, 41%, 40% and 19% for Co_3_O_4_/N-CP-900, respectively, 50%, 38%, and 12% for Co_3_O_4_/N-CP-1000, respectively. There results show that the proportions of graphitic-type quaternary N increased with increasing temperature, and the proportion of pyrrole N decreased due to its instability. Research shows that the content of graphitic-type quaternary N determines the limiting current density, while the pyridinic N content increased the ORR onset potential^29^. Pyridinic N contribute a *p*-electron to the aromatic *p*-system and has a single electron pair in the plane of carbon matrix, which can improve the electron donating ability. Thus, it weakens the O-O bond by bonding oxygen with nitrogen and/or adjacent carbon atoms, which facilitates the reduction of O_2_^30^. The optimum oxygen reduction performance of the Co_3_O_4_/N-CP-900 is due to the high content of graphitic-type quaternary N and pyridinic N^31,32^. The C1s XPS spectrum of Co_3_O_4_/N-CP-900 (Fig. S5d) can be deconvoluted into three peaks corresponding to graphene (284.0 eV)^33^, C-N (285.0 eV) and carboxyl C = O (288.4 eV) carbon bonded with oxygen, which corresponded to the O1s peak (Fig. S5e). The Co2p XPS spectrum of the Co_3_O_4_/N-CP-900 was given in Fig. 4e, and the data showed two major peaks at 780.0 (795.5) and 783.7 (797.4) eV corresponding to the Co and cobalt compounds (Co-O or Co-N)^34,35^. Taken together, we deduced that Co_3_O_4_/N-CP-X catalysts were composed of Co_3_O_4_ decorated N-doped carbon.

### Characterization by cyclic voltammetry

**Figure 5:**
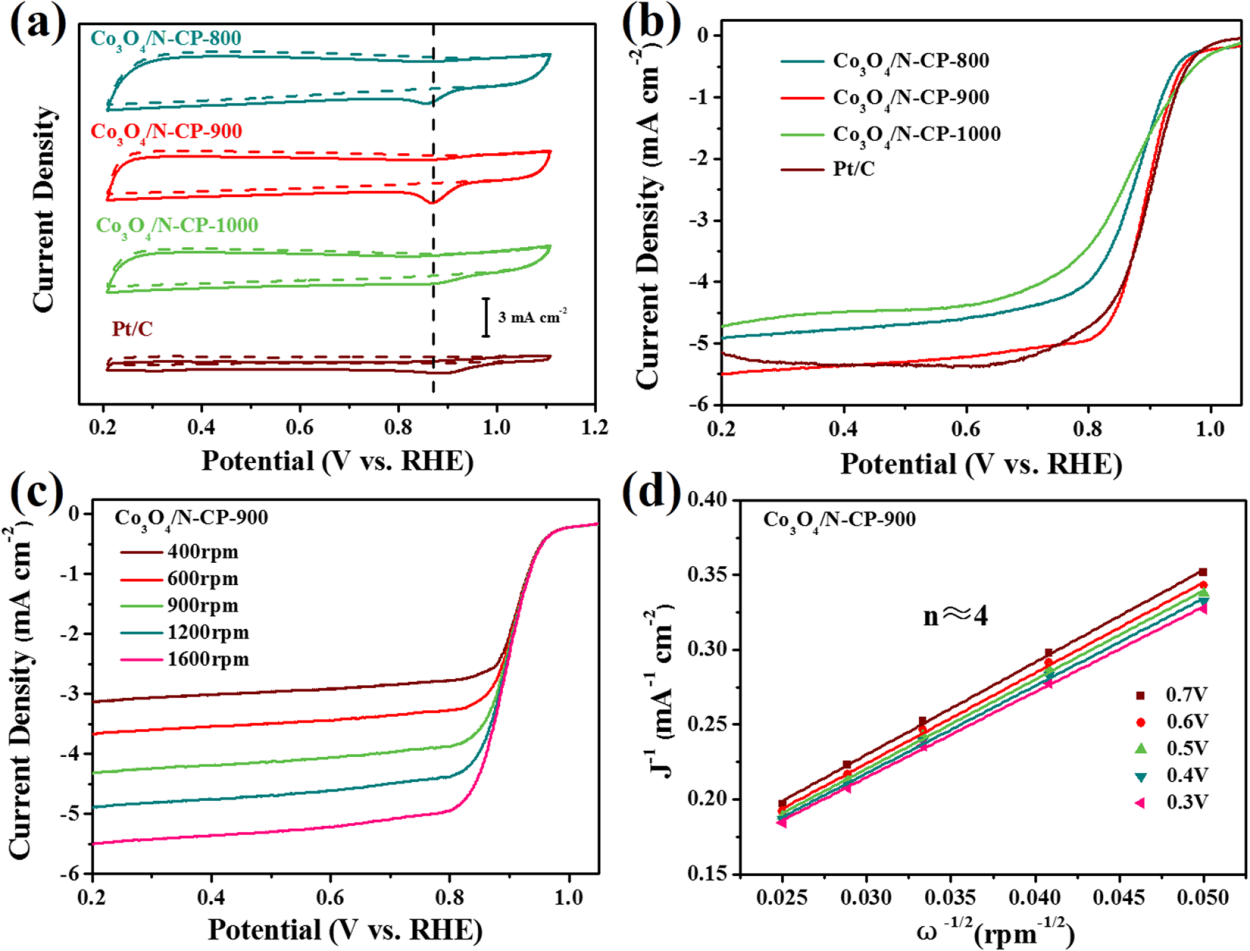
(**a**) CV curves of Co_3_O_4_/N-CP-800, Co_3_O_4_/N-CP-900, Co_3_O_4_/N-CP-1000 and commercial Pt/C in N_2_ (dotted lines) and O_2_-saturated (solid lines) 0.1 M KOH solution with a scan rate of 50 mV s^−1^. (**b**) LSV curves of Co_3_O_4_/N-CP-800, Co_3_O_4_/N-CP-900, Co_3_O_4_/N-CP-1000 and commercial Pt/C in O_2_-saturated 0.1 M KOH solution at a rotation rate of 1600 rpm with a scan rate of 10 mV s^−1^. (**c**,**d**) LSV curves and related K-L plots of Co_3_O_4_/N-CP-900 in O_2_-saturated 0.1 M KOH solution with a scan rate of 10 mV s^−1^ at different rotation rates from 400 to 1600 rpm.

The ORR electrocatalytic activities of coral-like Co_3_O_4_/N-CP-X and commercial Pt/C catalysts were evaluated by cyclic voltammetry (CV). As seen in Fig. 5a, all catalysts showed a quasi-rectangular voltammograms (dashed lines) without a redox peak over the potential range of 0.2 to 1.1 V in the N_2_-saturated solution. In the O_2_-saturated solution, the Co_3_O_4_/N-CP-900 catalysts observed an ORR reduction with peak potential at 0.88 V (vs. RHE), which was more positive than Co_3_O_4_/N-CP-800 (0.85 V) and Co_3_O_4_/N-CP-1000 (0.86 V), basically compared to Pt/C catalyst (0.90 V). Moreover, the Co_3_O_4_/N-CP-900 exhibited a higher peak current density (4.32 mA cm^−2^) than Pt/C catalyst (1.66 mA cm^−2^). The CV curves of N-CP-900 without Co_3_O_4_ were also measured for comparison. The CV curves in Fig. S6a show obvious oxygen reduction peak for N-CP-900 at 0.81 V. It is found that Co_3_O_4_/N-CP-900 exhibit a higher active than N-CP-900 catalyst, due to combination of cobalt oxide with N-doped carbon, which prove that Co species plays a key role in improving ORR performance. The above results showed that coral-like Co_3_O_4_/N-CP-900 catalyst is a high performance ORR electrocatalyst.

### Electrocatalytic reduction of oxygen

Then, we measured the linear sweep voltammogram (LSV) at a rotation rate of 1600 rpm on a rotating disk electrode (RDE). The LSV curves of Co_3_O_4_/N-CP-900 displayed the onset and half-wave potentials of 0.97 and 0.90 V in Fig. 5b. which was more positive than Co_3_O_4_/N-CP-800 (0.96 V and 0.87 V), Co_3_O_4_/N-CP-1000 (0.98 V and 0.86 V) and N-CP-900 (0.89 and 0.85 V) (Fig. S6b), and very similar to Pt/C catalyst (0.99 V and 0.89 V). The limited current density of Co_3_O_4_/N-CP-900 (5.50 mA cm^−2^) was comparable to Pt/C catalyst (5.15 mA cm^−2^). The ORR performances of cobalt/nitrogen-codoped carbon materials are summarized in Table S1. Compared with other reports, Co_3_O_4_/N-CP-900 exhibit excellent ORR performance, with more positive onset and half-wave potential, as well as a high diffusion-limited current density. In summary, these results indicate that the coral-like Co_3_O_4_/N-CP-900 catalyst exhibits excellent ORR catalytic activity, which can be used as a promising candidate material for commercial Pt/C catalysts.

LSV curves of Co_3_O_4_/N-CP-900, Co_3_O_4_/N-CP-800, Co_3_O_4_/N-CP-1000 and commercial Pt/C catalyst in O_2_-saturated 0.1 M KOH at different rotation rate were shown in Fig. 5c and Fig. S7a–c. RDE measured showed that the electron transfer number was close to 4.0 at 0.3–0.7 V on the basis of the Koutecky-Levich (K-L) plots (Fig. 5d, Fig. S7d–f). Compared to Co_3_O_4_/N-CP-800 and Co_3_O_4_/N-CP-1000, Co_3_O_4_/N-CP-900 was closer to a 4-electron transfer process toward ORR.

**Figure 6:**
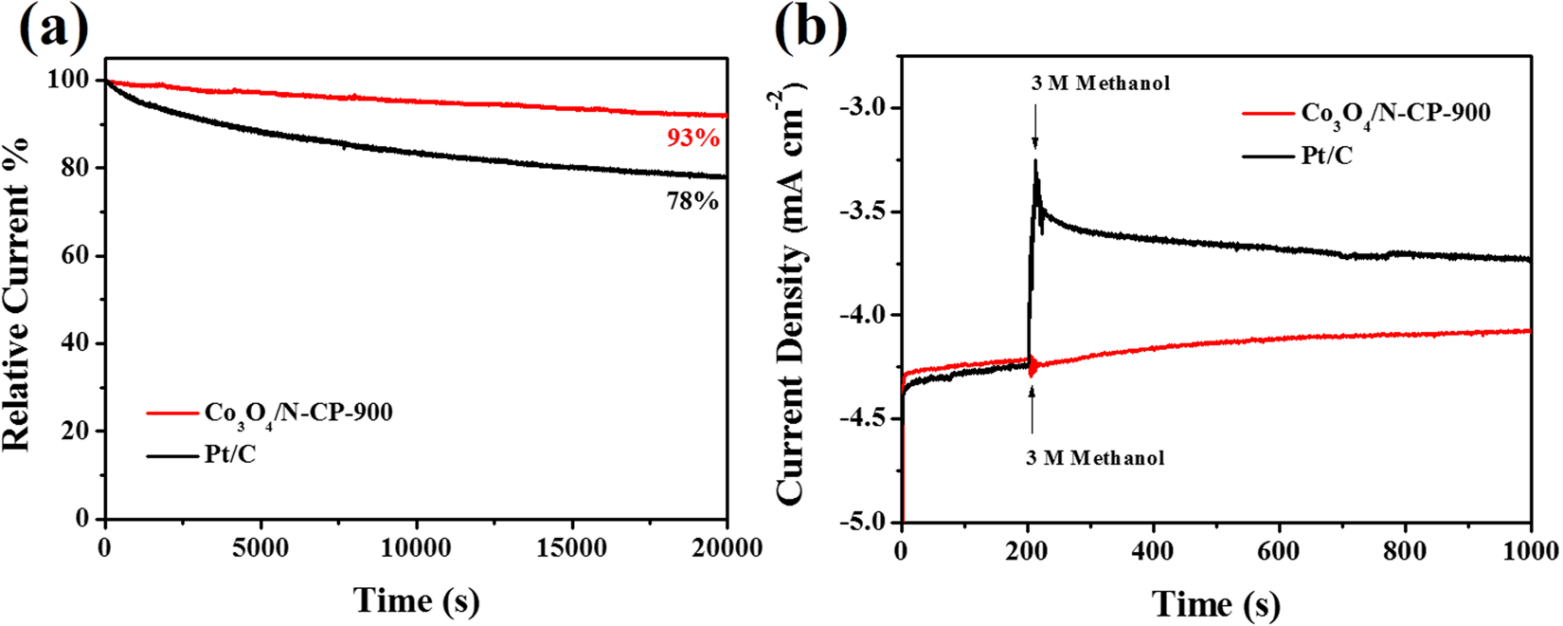
(**a**) Chrono-amperometric current-time (**i**–**t**) curves of Co_3_O_4_/N-CP-900 and commercial Pt/C in O_2_-saturated 0.1 M KOH solution at 0.4 V. b) Chrono-amperometric responses of Co_3_O_4_/N-CP-900 and commercial Pt/C at 0.4 V to adding 3.0 M methanol into O_2_-saturated 0.1 M KOH solution.

The stability of the catalyst is also a problem to be considered for ORR. We accelerated the degradation of Co_3_O_4_/N-CP-900 catalyst using the current-time (i-t) method. Co_3_O_4_/N-CP-900 catalyst exhibits such excellent durability, that it still has a high retention of 93% compared to the initial current even after 20000 s test, while only 78% of the commercial Pt/C catalyst was retained (Fig. 6a). In addition, we also tested the methanol tolerance of Co_3_O_4_/N-CP-900 catalyst, compared with commercial Pt/C catalyst. The current density of the Co_3_O_4_/N-CP-900 catalyst remained almost constant after the addition of 3 M methanol, whereas the commercial Pt/C catalyst showed a significant current change (Fig. 6b). In conclusion, coral-like Co_3_O_4_/N-CP-900 catalyst have higher stability and better methanol tolerance than commercial Pt/C catalyst.

### Electrochemical performance of supercapacitor

**Figure 7:**
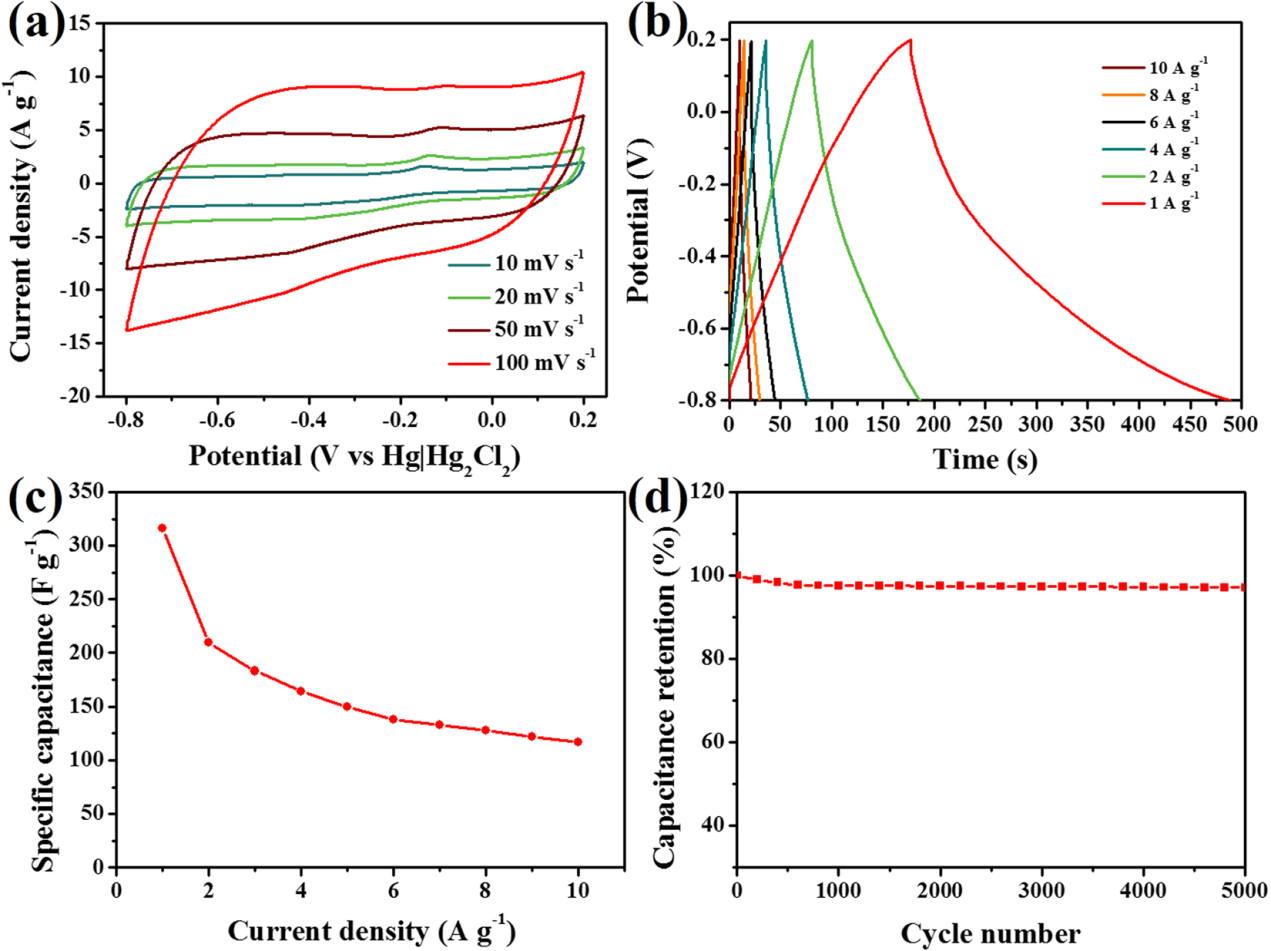
Electrochemical performance of supercapacitor by using a Co_3_O_4_/N-CP-900 electrode with a two-electrode system: (**a**) Cyclic voltammetry curves and (**b**) galvanostatic charge/discharge curves by using 6 M KOH as electrolyte, (**c**) Relationship between specific capacitances (C_s_) and current density, (**d**) Cycling stability of Co_3_O_4_/N-CP-900 at 5.0 A g^−1^ in 6 M KOH.

Except for the ORR catalyst, the coral-like Co_3_O_4_/N-CP-X material can be used as an electrode material for the energy storage device. We explored supercapacitor performance by assemble symmetrical two-electrode cell. Fig. 7a showed a typical CVs of a Co_3_O_4_/N-CP-900 electrode with a scanning rate of 10 to 100 mV s^−1^ at the potential range of -0.8 to 0.2 V in 6 M KOH aqueous solution, which was close to the rectangle, implying the characteristics of carbon based supercapacitors^36^. As the specific capacitance increases with current density, so the CV curve area is proportional to the specific capacitance. With the increase of scan rate, there is no obvious change of CV curves, indicating that the Co_3_O_4_/N-CP-900 electrode has fast charge and discharge performance^37^. At the same time, we compared the performance of samples obtained at different pyrolysis temperatures. The internal area of CV curve for Co_3_O_4_/N-CP-900 was enlarged compared to that of Co_3_O_4_/N-CP-800 and Co_3_O_4_/N-CP-1000, indicating that the specific capacitance of Co_3_O_4_/N-CP-900 is higher than that of Co_3_O_4_/N-CP-800 and Co_3_O_4_/N-CP-1000 (Fig. S8a). As can be seen from Fig. 7b, the galvanostatic charge and discharge curves were almost triangular, which approximated the ideal supercapacitors behavior. The results showed that the high specific surface area and pore volume of Co_3_O_4_/N-CP-900 results in excellent supercapacitor performance. As shown in Fig. 7c, the specific capacitance (C_s_) at 1.0 A g^−1^ in the 6 M KOH aqueous solution was calculated to be 316.2 F g^−1^ by the galvanostatic discharge curve, and the C_s_ values also reached 117.1 F g^−1^ even at the current density of 10 A g^−1^. The microporous structure results in high specific surface area and pore volume, leading to Co_3_O_4_/N-CP-900 with a good capacitance. Specific capacitances are 225.5, 316.2 and 174.6 F g^−1^ at a current density of 1.0 A g^−1^ for the samples Co_3_O_4_/N-CP-800, Co_3_O_4_/N-CP-900 and Co_3_O_4_/N-CP-1000, respectively (Fig. S8b). The results were consistent with the area of CV curves. As the pyrolysis temperature increases, the specific surface area of the electrode material increases gradually, resulting in a higher specific capacitance of Co_3_O_4_/N-CP-900 than that of Co_3_O_4_/N-CP-800. However, the specific capacitance of Co_3_O_4_/N-CP-1000 is significantly lower than that of Co_3_O_4_/N-CP-900, which is due to the too high pyrolysis temperature, causing the electrode material to form clumps and the electrolyte unable to penetrate into the electrode material. We also performed galvanostatic charge and discharge tests at a current density of 5 A g^−1^ to evaluate the durability of the Co_3_O_4_/N-CP-900 electrode. After 5000 cycles of charge and discharge, a high capacity retention of 90% was achieved, showing good cycle performance (Fig. 7d). The excellent performance of Co_3_O_4_/N-CP-900 electrode is due to the uniform N doping and Co_3_O_4_ anchoring effect, which improves the conductivity, wettability and reactivity of the materials.

**Figure 8:**
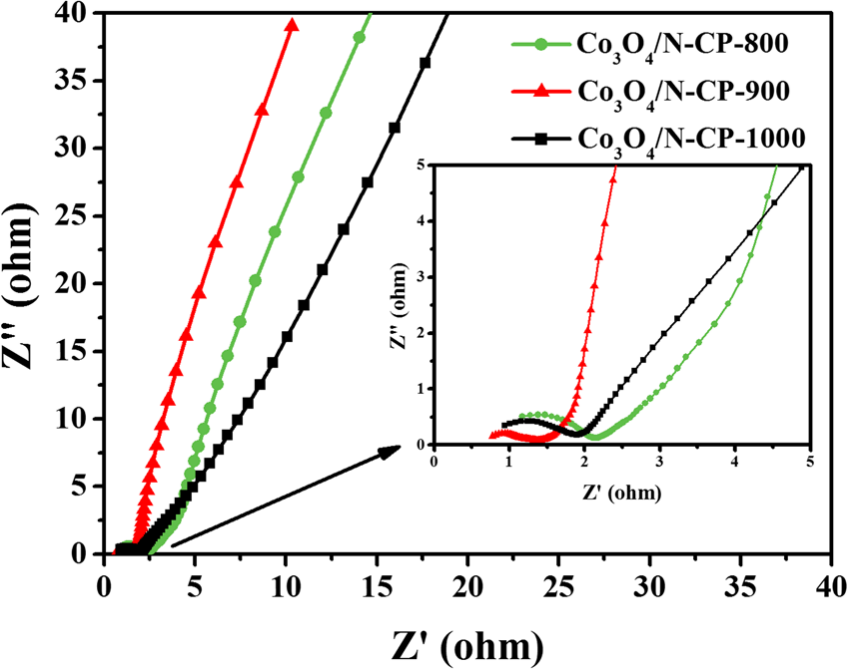
Electrochemical impedance spectrum of the Co_3_O_4_/N-CP-800, Co_3_O_4_/N-CP-900 and Co_3_O_4_/N-CP-1000 electrode in 6 M KOH electrolyte. The inset shows the magnified electrochemical impedance spectrum in a high-frequency range.

In addition, we characterized the electrochemical impedance spectroscopy (EIS) of Co_3_O_4_/N-CP-800, Co_3_O_4_/N-CP-900 and Co_3_O_4_/N-CP-1000 at room temperature. The Nyquist plot of Co_3_O_4_/N-CP-900 electrode was obtained in 6 M KOH aqueous solution (Fig. 8). From the high-frequency end of the Nyquist plot x intercept can be 0.75 Ω resistance, which is almost the resistance of the electrolyte^38^. Co_3_O_4_/N-CP-900 showed a smaller kinetic arc at high-frequencies than that of Co_3_O_4_/N-CP-800 and Co_3_O_4_/N-CP-1000, which means that its charge transfer resistance was relatively lower than that of Co_3_O_4_/N-CP-800 and Co_3_O_4_/N-CP-1000, indicating that the charge transfer efficiency of Co_3_O_4_/N-CP-900 was high. The low frequency stimulation of the EIS spectrum of Co_3_O_4_/N-CP-900 was almost vertical, which indiacated that the ions rapidly diffused in the electrolyte during charge and discharge^14^. This shows that Co_3_O_4_/N-CP-900 is closer to the ideally capacitive behavior than Co_3_O_4_/N-CP-800 and Co_3_O_4_/N-CP-1000. The above test results show that the supercapacitor with Co_3_O_4_/N-CP-900 electrode has extremely low internal resistance. We conclude that the good electrochemical performance of coral-like Co_3_O_4_/N-CP-900 electrode can be attributed to the combined effect of high specific surface area, narrow pore size distribution and heteroatom doping. The interconnected microporous structure allows the electrolyte to pass more efficiently and reduces the resistance within the electrode. Moreover, heteroatom doping can increase the surface wettability, pseudocapacitance, chemical stability and conductivity of carbon materials.

## Conclusions

In summary, a coral-like Co_3_O_4_/N-CP with high electrochemical performances toward ORR and supercapacitor electrode in alkaline media was fabricated by chemical polymerization of aniline, hydrothermal, and pyrolysis. Co_3_O_4_/N-CP-900 showed a positive onset potential of 0.97 V, half-wave potential of 0.90 V, as well as a high diffusion-limited current density of 5.50 mA cm^−2^ in 0.1 M KOH. Compared to Pt/C catalysts, it also exhibits good stability and excellent methanol tolerance. In addition, Co_3_O_4_/N-CP-900 electrode exhibited an excellent specific capacitance of 316.2 F g^−1^ in 6 M KOH aqueous solution at a current density of 1.0 A g^−1^, as well as good rate capabilities and high cycling stabilities. The good activity of ORR and supercapacitors is due to the high specific surface area and microporous structure, which not only improves the availability of electron transport within the surface area, but also allows the reactants to be better delivered. With the decoration of Co_3_O_4_ on N-doped carbon, the ORR catalytic activity could be improved significantly due to a high density of active sites. This work may provide an approach to develop transition metal oxide decorated nitrogen co-doped carbon materials as advanced catalysts for use in electrochemical energy conversion and storage.

## Methods

### Materials

Aniline was purchased from Aladdin, and distilled before use. Perfluorosebacic acid was purchased from TCI(Shanghai) Development Co., Ltd. Co(NO_3_)_2_·6H_2_O, KOH, ammonium persulfate, methanol, and ether were purchased from Beijing Beihua Fine Chemicals Co., Ltd. Commercial carbon-supported Pt catalyst (20 wt.%, Pt/C) was purchased from Sigma-Aldrich. Nafion (DuPont, 10 wt.%) was diluted to 0.05 wt% using ethanol.

### Synthesis of coral-like Co_3_O_4_ decorated N-doped carbon particles (Co_3_O_4_/N-CP)

First, 20 mL of 0.025 mol L^−1^ perfluorodecanoic acid (PFSEA) aqueous solution was prepared, and then 4 mmol aniline was added to form a uniform emulsion under ultrasonic action. Subsequently, 20 mL of 0.2 mol L^−1^ ammonium persulfate (APS) aqueous solution was added to the above uniform emulsion and reacted at 12 °C for 15 hours. In order to completely remove the by-product, the obtained polyaniline was washed with methanol, ether and deionized water, then filtered and dried to obtained coral-shaped polyaniline (PANI) nanoparticles. Co_3_O_4_/PANI was prepared by hydrothermal method, as reported previously^23^. The obtained polyaniline was added to 40 mL deionized water under ultrasonic conditions, and 4 mmol Co(NO_3_)_2_·6H_2_O was added to the above solution. After 30 min of sonication, the mixture was added to a 50 mL polytetrafluoroethylene liner stainless autoclave, then heated at 180 °C for 24 h. The Co_3_O_4_/PANI was collected by centrifugation and washed with deionized water and ethanol. Finally, the materials were prepared by pyrolyzing the precursors at 800 °C, 900 °C, 1000 °C in a nitrogen atmosphere for 3.0 h at a heating rate of 5 °C/min. The final product was denoted as Co_3_O_4_/N-CP-X (X represents temperature). For comparison, the metal-free carbon catalyst (referred to as N-CP-X) was also prepared by the same procedure as that of Co_3_O_4_/N-CP-X, except that cobalt nitrate was removed during the hydrothermal reaction.

### Characterization

The microstructure of the Co_3_O_4_/N-CP-X were observed by field emission scanning electron microscope (FESEM) (JEOL JSM-7500F), environmental scanning electron microscopy (ESEM) (Quanta 250 FEG) and field emission transmission electron microscope (TEM) (JEOL JEM-2100F). X-ray diffraction (XRD) were conducted using the PANalytical, Empyrean XRD (CuKa 1.5406 Å radiation), and the surface chemical composition of the Co_3_O_4_/N-CP-X was obtained by the X-ray photoelectron spectroscopy (XPS) (VG ESCALAB 220i-XL photoelectron spectrometer with a monochromatic AlKα X-ray source). The surface area (BET) and pore size distribution (BJH) were performed on Micromeritics ASAP 2020 V3.00 H system. The Raman spectra was recorded on a Laboratory RAM HR1800.

### Oxygen reduction reaction measurements

Oxygen reduction reaction properties were measured using a three-electrode system on the CHI 760D (Chenhua, Shanghai) electrochemical workstation. Preparation of working electrode by weighing 2 mg of catalyst into 1 mL of ethanol solution, ultrasonic 20 min, forming a highly dispersed catalyst solution (2 mg mL^−1^). Take 15 uL of the catalyst ethanol solution into the rotating disk electrode (RDE, disc diameter 3 mm) surface, room temperature drying, and then dropping 7.5 uL 0.05% Nafion ethanol solution, room temperature drying. Among them, the platinum wire and the saturated Hg|Hg_2_Cl_2_ (KCl sat.) were used as the counter electrode and the reference electrode, respectively, and the rotating disk electrode with the catalyst was used as the working electrode. The cyclic voltammetry (CV) tests were -carried out in O_2_-saturated and N_2_-saturated 0.1 mol L^−1^ KOH solution with a scanning rate of 50 mV s^−1^ at a test potential ranging from -0.8 V to 0.1 V, and linear sweep voltammetry (LSV) tests at a scanning rate of 10 mV s^−1^ at different rotational rates of 400, 600, 900, 1200 and 1600 rpm in 0.1 mol L^−1^ KOH solution saturated with O_2_. All tests were carried out in a 25 °C thermostatic system.

The number of electrons transferred in the ORR process (n) is determined using the Koutecky-Levich (K-L) (1):

1 $$\begin{array}{c}\frac{1}{J}=\frac{1}{{J}_{K}}+\frac{1}{{J}_{L}}=\frac{1}{{J}_{K}}+\frac{1}{B{\omega }^{1/2}}\\ B=0.2\,nF{C}_{{O}_{2}}{({D}_{{O}_{2}})}^{2/3}{v}^{-1/6}\end{array}$$

Here, *J*, *J*_*K*_ and *J*_*L*_ are the measured current density, kinetic current density and diffusion-limited current density, respectively, ω is the rotation rate, *B* can be determined by the slope of K-L plots based on the Levich equation, *n* is the number of electrons transferred per oxygen molecule, *F* is the Farady constant (96485 C mol^−1^),$\,{C}_{{O}_{2}}$ is the bulk concentration of O_2_ in 0.1 mol L^−1^ KOH (1.2 × 10^−6^ mol cm^−3^), ${D}_{{O}_{2}}$ is the diffusion coefficient of O_2_ in 0.1 mol L^−1^ KOH (1.9 × 10^−5^ cm^2^ s^−1^) and *υ* is the kinetic viscosity (1.10 × 10^−2^ cm^2^ s^−1^)^39,40^.

The electrode stability at the bias potential of 0.8 V (vs. RHE) in O_2_-saturated 0.1 M KOH solutions using current-time (i-t) method with a rotation rate of 1000 rpm, Then, 5 mL methanol is added to the O_2_-saturated 0.1 M KOH aqueous solution to test the tolerance to methanol crossover effect.

### Supercapacitor measurements

Supercapacitor properties were measured using a three electrode cell on the CHI 760E (Chenhua, Shanghai) electrochemical workstation. For capacitance measurements, the Co_3_O_4_/N-CP-900 particles, polyvinylidene fluoride, and carbon black were mixed at a weight ratio 8:1:1 in N-Methyl-2-pyrrolidone solvent as the working electrode. The mixture was firstly well mixed to form a slurry and it was coated onto a piece of carbon paper (1 × 1 cm^2^), and dried for 12 h at 120 °C. The CV test at a scan rate of 10 to 100 mV s^−1^. Measure galvanostatic charge and discharge to evaluate specific capacitance and cyclability. The cells were charged/discharged at current rates ranging from 1 to 10 A g^−1^ for 10 cycles to measure the capacitance. The cells were charged/discharged at a current density of 5 A g^−1^ for 5000 cycles to tests the cyclability. The equation is used to calculate the specific capacitance (C_s_) of the electrode material (2):

2 $${C}_{s}=\frac{I{\rm{\Delta }}t}{{\rm{\Delta }}Vm}$$

Here, *I*, *∆t* and *m* are the discharge current, discharge time and mass of carbon on the electrode, respectively, *∆V* is the voltage difference within *∆t*. At the open circuit voltage, electrochemical impedance spectroscopy (EIS) test in the 10 mHz to 100 kHz frequency range, the amplitude of 5 mV. According to the constant current discharge process with different current density, the ragone diagram was calculated^38,41^.

## Electronic supplementary material

Supplementary Information
