# Supplementary material for: Coral-like Co3O4 Decorated N-doped Carbon Particles as active Materials for Oxygen Reduction Reaction and Supercapacitor
Source: Sci Rep. 2018 Jan 29;8:1802. doi: 10.1038/s41598-018-19347-5 (PMC5789056; doi:10.1038/s41598-018-19347-5)
Supplement: Supplementary file 1 — Supplementary Information [file 41598_2018_19347_MOESM1_ESM.docx]

**Supplementary information**

**Coral-like Co_3_O_4_ Decorated N-doped Carbon Particles as active Materials for Oxygen Reduction Reaction and Supercapacitor**

**Zhichao Lin^1^, Xiuwen Qiao^1^***

^1^Key Laboratory for Green Processing of Chemical Engineering of Xinjiang Bingtuan, State Key Laboratory Cultivation Base Jointly Constructed by Province and The Ministry, Key Laboratory of Materials-Oriented Chemical Engineering of Xinjiang Uygur Autonomous Region, College of Chemistry and Chemical Engineering, Shihezi University, Shihezi,832000,China (email: linzc@stu.shzu.edu.cn)


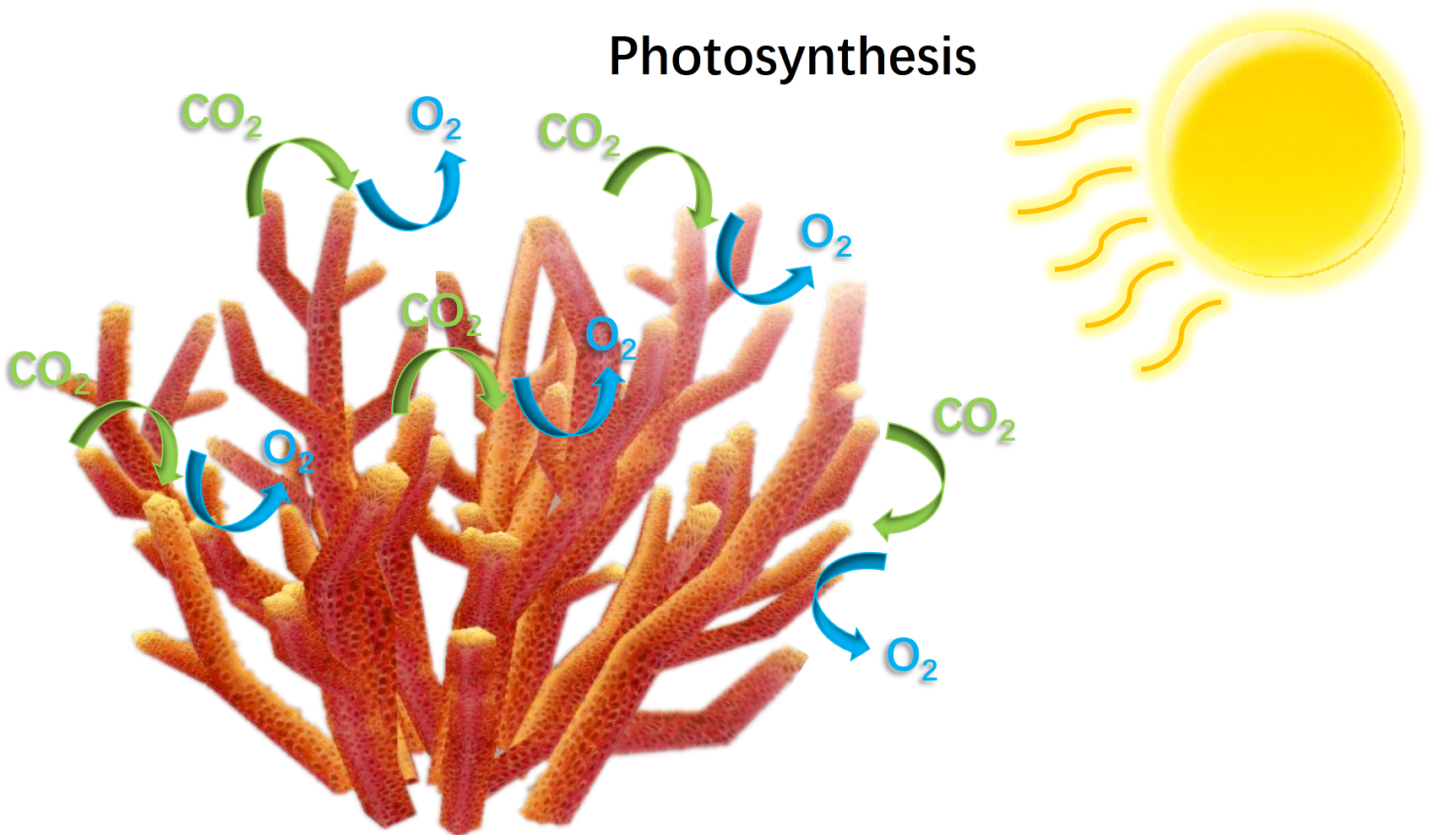


**Figure S1.** Gas exchange in coral ecosystems.


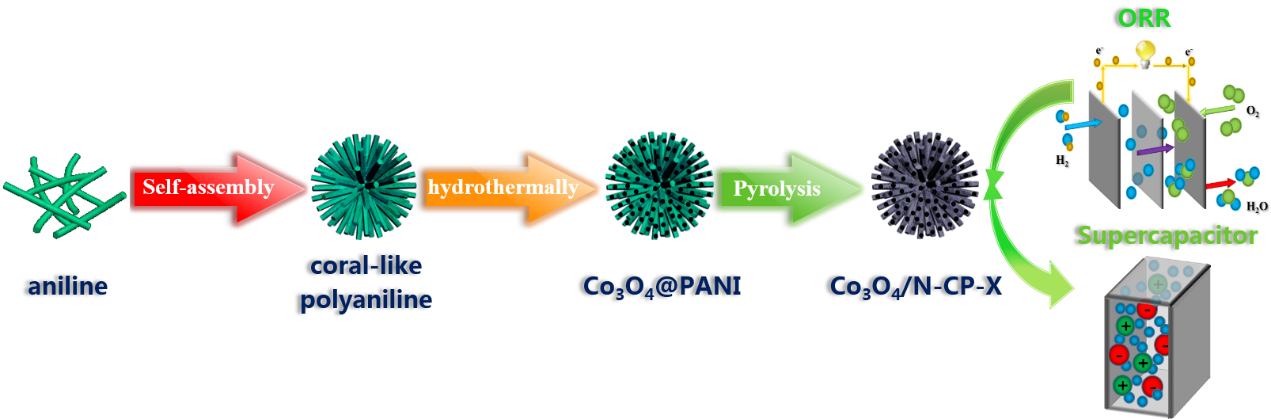


**Figure S2.** The Co_3_O_4_/N-CP-X fabricated by pyrolysis of the self-assembled Co_3_O_4_/PANI precursor displayed efficient catalytic activity for oxygen reduction and excellent capacitive properties in an alkaline solution.

**
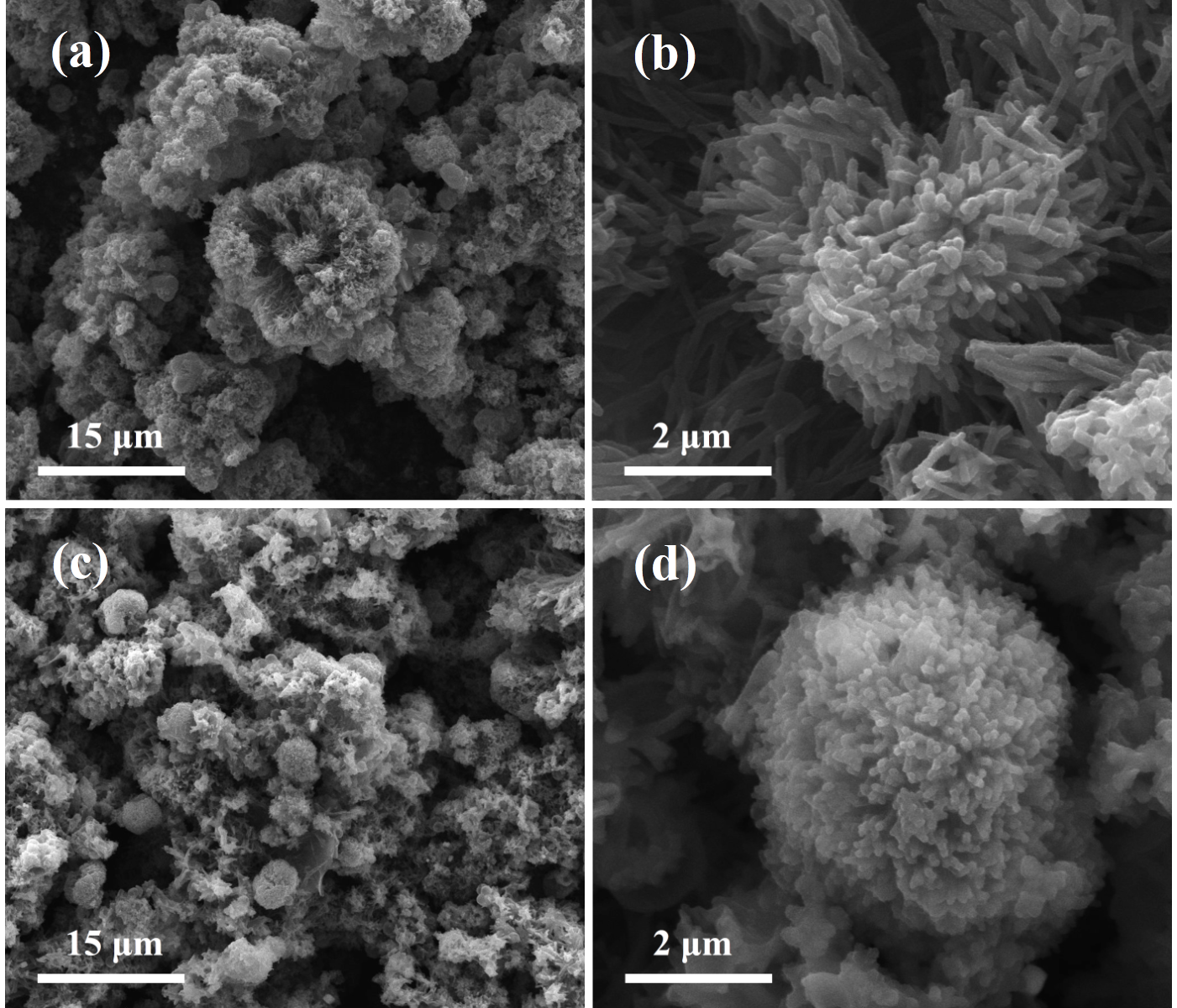
**

**Figure S3.** a-b) SEM images of the coral-like polyaniline, c-d) SEM images of Co_3_O_4_@PANI.

**
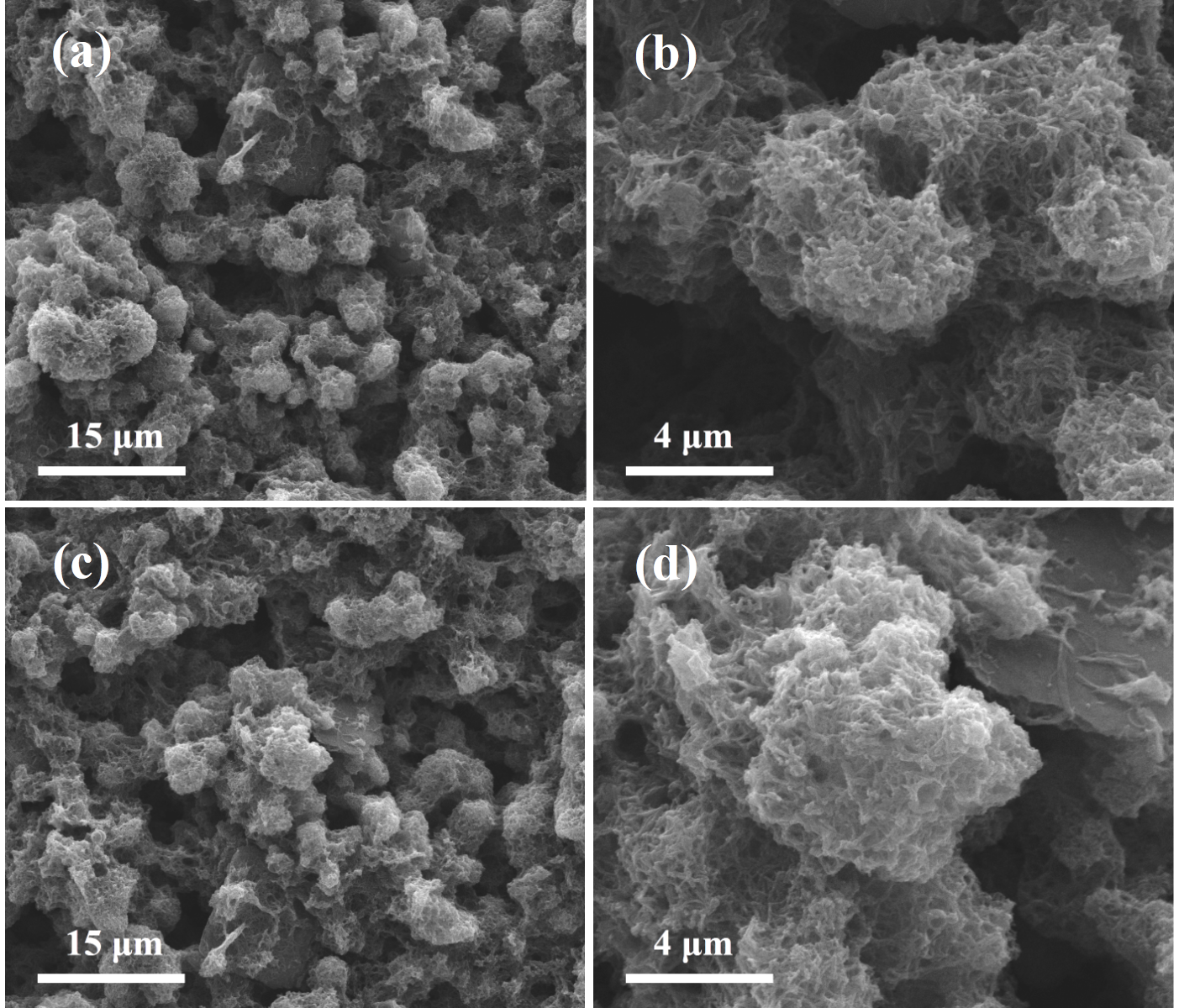
**

**Figure S4.** a-b) SEM images of Co_3_O_4_/N-CP-800, c-d) SEM images of Co_3_O_4_/N-CP-1000.


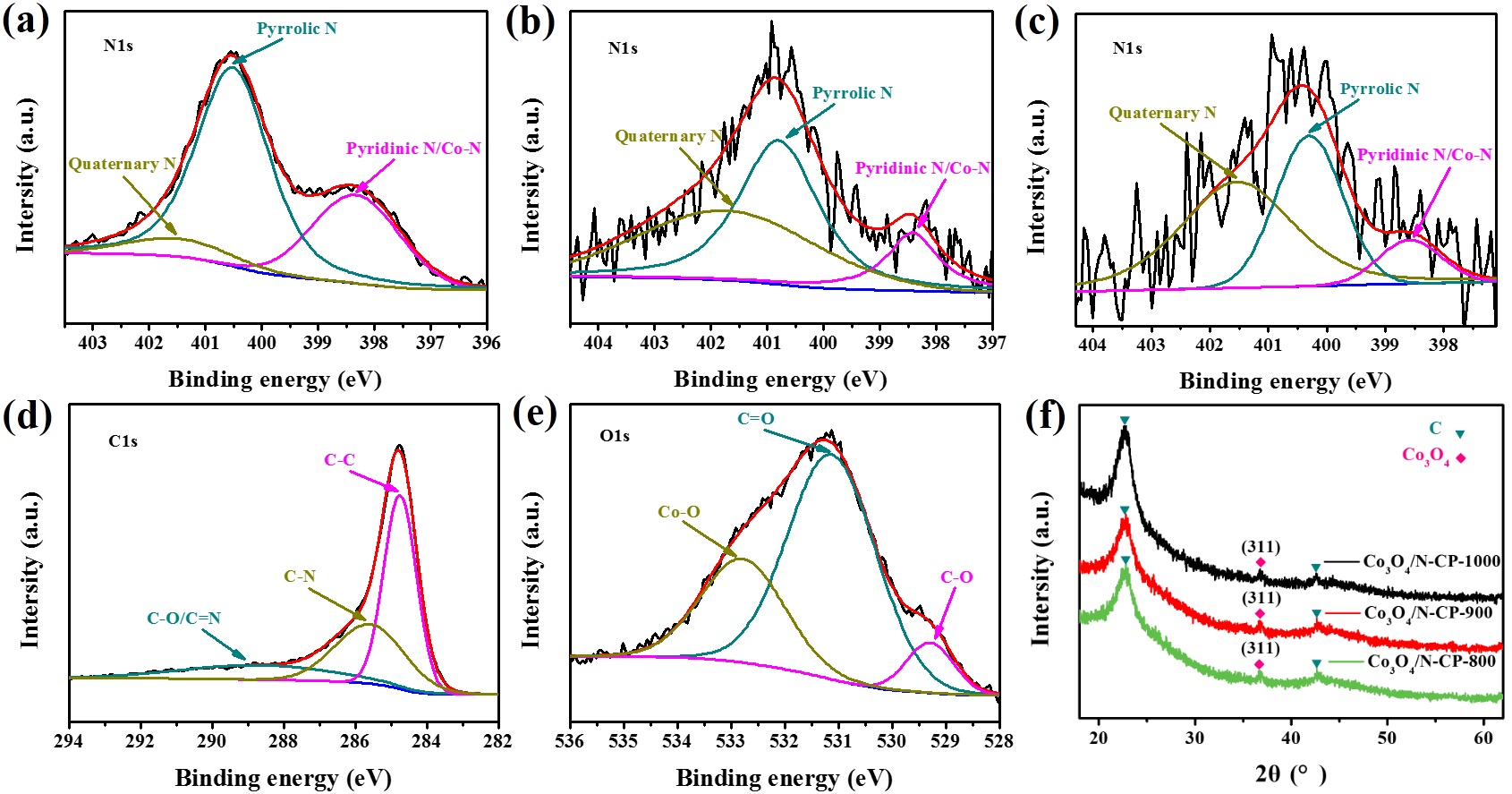


**Figure S5.** High-resolution N1s XPS spectrum of the a) Co_3_O_4_/N-CP-800, b) Co_3_O_4_/N-CP-900 and c) Co_3_O_4_/N-CP-1000, d, e) High-resolution C1s and O1s XPS spectrum of the Co_3_O_4_/N-CP-900, f) XRD of the Co_3_O_4_/N-CP-800, Co_3_O_4_/N-CP-900 and Co_3_O_4_/N-CP-1000.


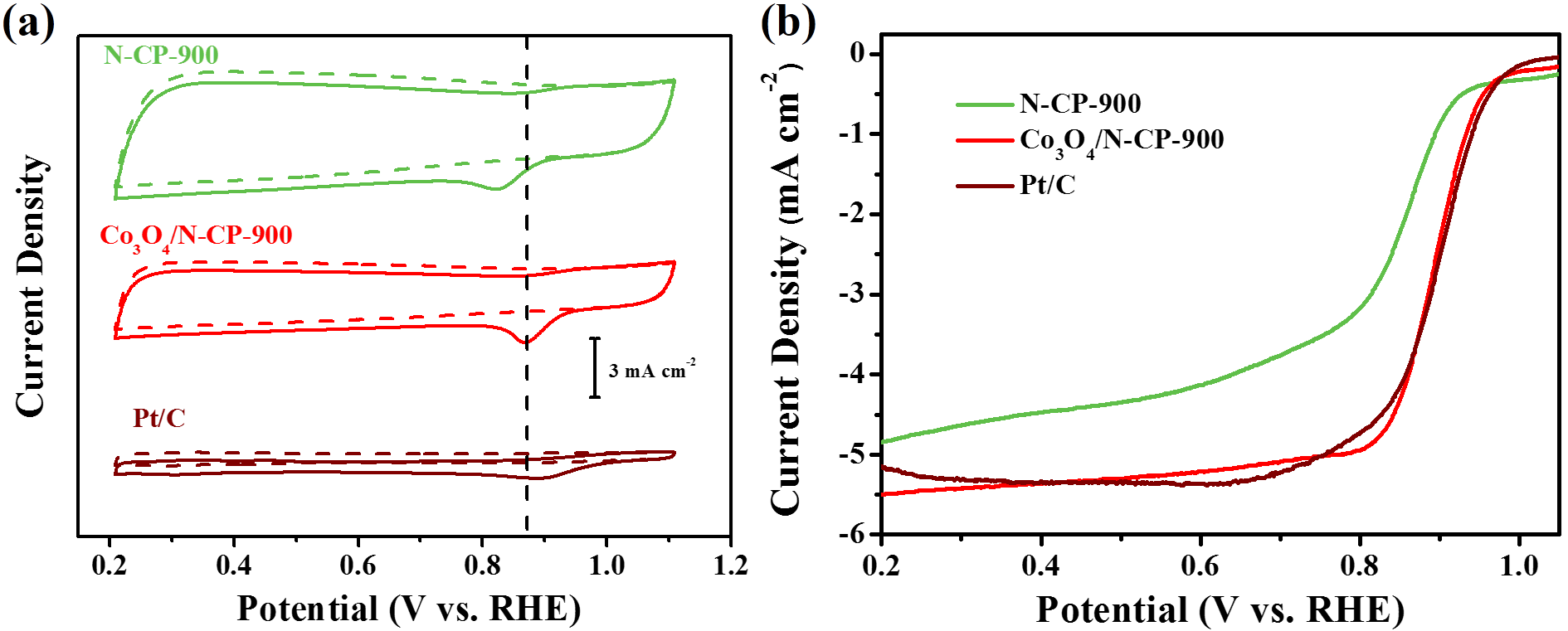


**Figure S6.** a) CV curves of N-CP-900, Co_3_O_4_/N-CP-900 and commercial Pt/C in N_2_ (dotted lines) and O_2_-saturated (solid lines) 0.1 M KOH solution with a scan rate of 50 mV s^-1^, b) LSV curves of N-CP-900, Co_3_O_4_/N-CP-900 and commercial Pt/C at a rotation rate of 1600 rpm with a scan rate of 10 mV s^-1^.


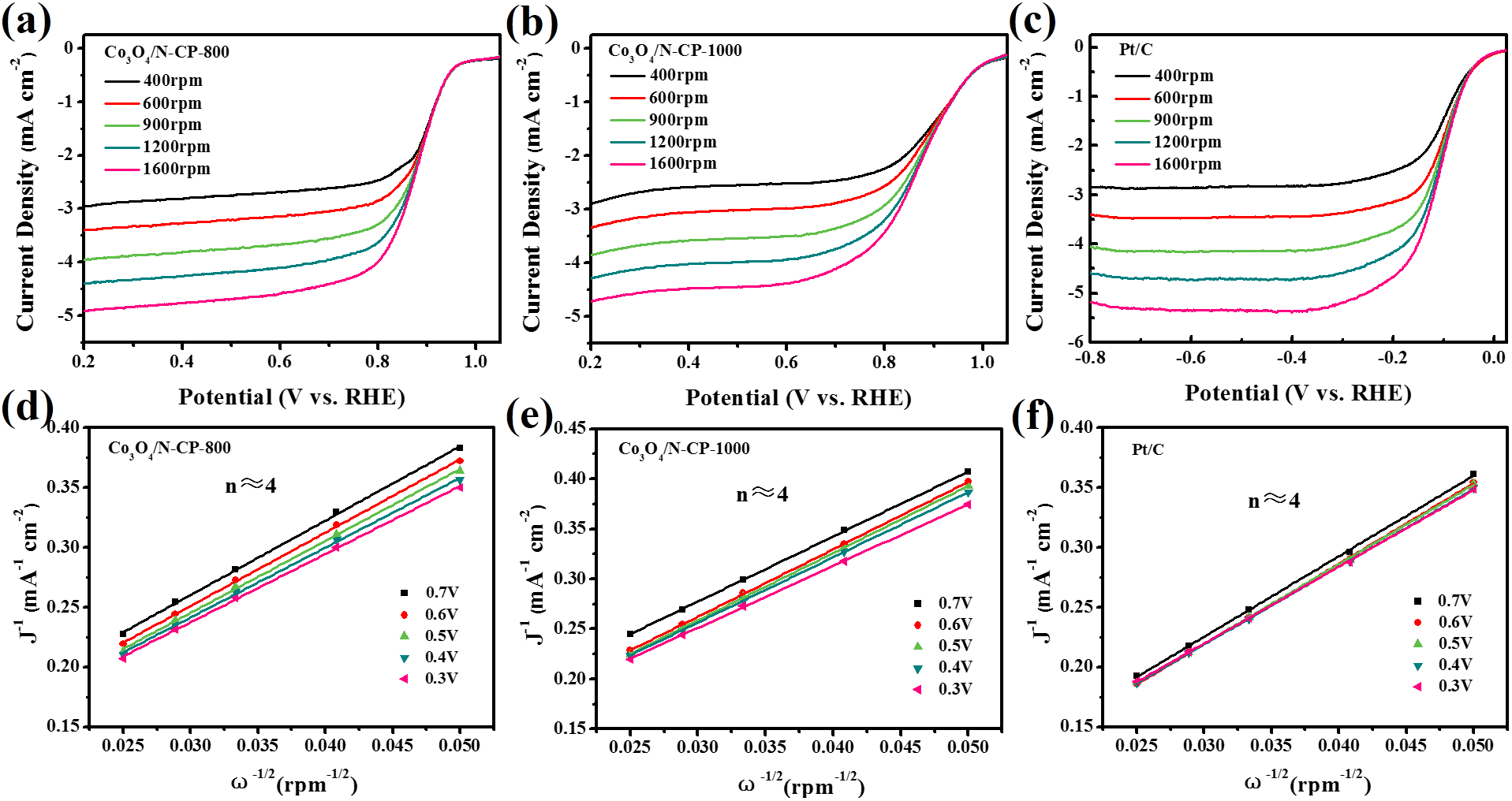


**Figure S7.** LSV curves and related K–L plots of a, d) Co_3_O_4_/N-CP-800, b, e) Co_3_O_4_/N-CP-1000, and c, f) commercial Pt/C catalysts in O_2_-saturated 0.1 M KOH solution at a scan rate of 10 mV s^-1^ at different rotation rates from 400 to 1600 rpm.


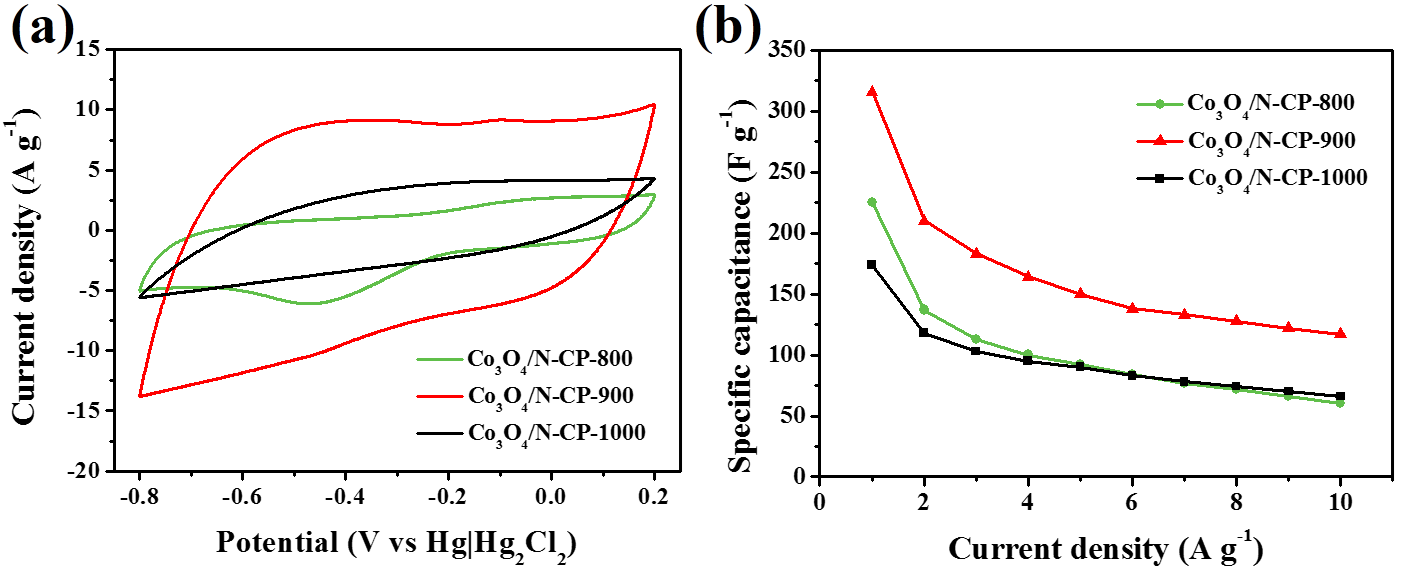


**Figure S8.** a) Cyclic voltammetry curves with a scanning rate of 100 mV s^-1^, b) Relationship between specific capacitances (C_s_) and current density of Co_3_O_4_/N-CP-800, Co_3_O_4_/N-CP-900 and Co_3_O_4_/N-CP-1000 electrode in 6 M KOH.

**Table S1.** ORR performances of cobalt/nitrogen-codoped carbon materials in O_2_ saturated 0.1 M KOH solution at a rotation rate of 1600 rpm

| **Electrocatalyst** | **Onset potential**  **(V vs. RHE)** | **Half-wave** **potential**  **(V vs. RHE)** | **Limiting current**  **density**  **(mA cm^-2^)** | **Ref** |
| --- | --- | --- | --- | --- |
| Co_3_O_4_/N-CP-800  Co_3_O_4_/N-CP-900  Co_3_O_4_/N-CP-1000 | 0.96  0.97  0.98 | 0.87  0.90  0.86 | 4.95  5.50  4.80 | This work |
| Pt/C | 0.99 | 0.89 | 5.15 | This work |
| Co_3_O_4_/N-rmGO | 0.90 | 0.83 | 5.00 | 1 |
| Co/CoO@Co-N-C-700  Co/CoO@Co-N-C-800  Co/CoO@Co-N-C-900  Co/CoO@Co-N-C-1000 | 0.90  0.93  0.89  0.87 | 0.75  0.81  0.76  0.75 | 4.85  5.60  4.85  5.30 | 2 |
| Co_3_O_4_-PPy/GN | 0.86 | 0.78 | 4.47 | 3 |
| CoO@NS-CSs | 0.95 | 0.82 | 5.50 | 4 |
| g-VB12(Co/N-Codoped) | 0.91 | 0.83 | 5.50 | 5 |

1. Liang, Y. et al. Co_3_O_4_ nanocrystals on graphene as a synergistic catalyst for oxygen reduction reaction. *Nat Mater.* **10**, 780-786 (2011)

2. Zhang, X. et al. Co/CoO nanoparticles immobilized on Co-N-doped carbon as trifunctional electrocatalysts for oxygen reduction, oxygen evolution and hydrogen evolution reactions. *Chem Commun*. **52**, 5946-5949 (2016)

3. Ren, G. et al. A bio-inspired Co_3_O_4_-polypyrrole-graphene complex as an efficient oxygen reduction catalyst in one-step ball milling. *Nano Res*. **8**, 3461-3471 (2015)

4. Chen, L., Guo, X., Zhang, G. N, S co-doped carbon spheres with highly dispersed CoO as nonprecious metal catalyst for oxygen reduction reaction. *Journal of Power Sources*. **360**, 106-113 (2017)

5. Jiang, Y. et al. A cobalt-nitrogen complex on N-doped three-dimensional graphene framework as a highly efficient electrocatalyst for oxygen reduction reaction. *Nanoscale*. **6**, 15066-15072 (2014)
